# Aldosterone signaling regulates the over-expression of claudin-4 and -8 at the distal nephron from type 1 diabetic rats

**DOI:** 10.1371/journal.pone.0177362

**Published:** 2017-05-11

**Authors:** Eduardo Molina-Jijón, Rafael Rodríguez-Muñoz, Ricardo González-Ramírez, Carmen Namorado-Tónix, José Pedraza-Chaverri, Jose L. Reyes

**Affiliations:** 1 Department of Physiology, Biophysics and Neuroscience, Center for Research and Advanced Studies of the National Polytechnic Institute (CINVESTAV-IPN), Mexico City, México; 2 Departamento de Biociencias e Ingeniería, Centro Interdisciplinario de Investigaciones y Estudios sobre el Medio Ambiente y Desarrollo del Instituto Politécnico Nacional (CIIEMAD-IPN), Mexico City, México; 3 Department of Molecular Biology and Histocompatibility, Dr. Manuel Gea González, General Hospital, Mexico City, México; 4 Department of Biology, Faculty of Chemistry, National Autonomous University of Mexico (UNAM), Mexico City, México; University of Louisville, UNITED STATES

## Abstract

Hyperglycemia in diabetes alters tight junction (TJ) proteins in the kidney. We evaluated the participation of aldosterone (ALD), and the effect of spironolactone (SPL), a mineralocorticoid receptor antagonist, on the expressions of claudin-2, -4, -5 and -8, and occludin in glomeruli, proximal and distal tubules isolated from diabetic rats. Type 1 diabetes was induced in female Wistar rats by a single tail vein injection of streptozotocin (STZ), and SPL was administrated daily by gavage, from days 3–21. Twenty-one days after STZ injection the rats were sacrificed. In diabetic rats, the serum ALD levels were increased, and SPL-treatment did not have effect on these levels or in hyperglycemia, however, proteinuria decreased in SPL-treated diabetic rats. Glomerular damage, evaluated by nephrin and Wilm’s tumor 1 (WT1) protein expressions, and proximal tubular damage, evaluated by kidney injury molecule 1 (Kim-1) and heat shock protein 72 kDa (Hsp72) expressions, were ameliorated by SPL. Also, SPL prevented decrement in claudin-5 in glomeruli, and claudin-2 and occludin in proximal tubules by decreasing oxidative stress, evaluated by superoxide anion (O_2_^●―^) production, and oxidative stress markers. In distal tubules, SPL ameliorated increase in mRNA, protein expression, and phosphorylation in threonine residues of claudin-4 and -8, through a serum and glucocorticoid-induced kinase 1 (SGK1), and with-no-lysine kinase 4 (WNK4) signaling pathway. In conclusion, this is the first study that demonstrates that ALD modulates the expression of renal TJ proteins in diabetes, and that the blockade of its actions with SPL, may be a promising therapeutic strategy to prevent alterations of TJ proteins in diabetic nephropathy.

## Introduction

The kidney plays a vital role in electrolyte homeostasis by mediating transepithelial reabsorption of water and ions, thereby determining the final composition of urine. Transepithelial transport can be either, transcellular that involves the passage through cells, mediated by transmembrane channels, transporters, and pumps or paracellular, which is the passage of substances between adjacent epithelial cells. The paracellular pathway is a route for passive transport [1], with the tight junction (TJ) constituting the primary permeability barrier [2]. Claudins (cldns) are TJ proteins that regulate the paracellular permeability of the renal epithelium to small solutes, and water [3]. Specific sets of cldns are expressed along of different nephron segments (glomeruli and tubules), thus determining unique segmental paracelular permeability. Glomeruli express cldns, such as claudin-1, in parietal epithelial cells [4,5], and claudin-5 and -6 in capillaries [6–8]. Decreased glomerular expression of cldn-5 has been associated with diabetes-induced proteinuria [9]. In renal tubules, pore cldns, such as claudin-2, are associated with leakier segments, such as the proximal tubule, whereas barrier cldns, such as claudin-4 and -8, are expressed in the aldosterone-sensitive distal nephron (ASDN) [10]. Cldn-2 is considered a paracellular pore, which participates in water, and sodium reabsorption, in the proximal tubule [11]. Cldns-4 and -8 act as cation barriers when overexpressed in MDCK cells [12–15], and both proteins may function primarily as paracellular chloride (Cl^―^) pores [16]. Occludin (occldn) is another integral protein of the TJ, which has been located at the proximal tubule, but is more abundant in the distal segments from nephron [17].

We previously studied the impact of diabetes on renal cldns expressions, and described that diabetes decreases the expression of cldn-5 in glomeruli (GL), and cldn-2 and occldn in proximal tubules (PT), in an oxidative stress-dependent way. In contrast, in distal tubules (DT), where oxidative stress was not observed, increased expressions of cldns-4 and -8 were found. The mechanisms responsible of these changes have not been explored [9].

Under diabetic conditions, the increased activity of the renin-angiotensin-aldosterone system (RAAS), leads to aldosterone (ALD) over-production. This increment in ALD, and the consequent activation of the mineralocorticoid receptor (MR), is associated to diabetic nephropathy development [18,19].

In addition to its physiological role in regulating renal sodium (Na^+^) reabsorption, and potassium (K^+^) excretion in the ASDN, ALD promotes tissue inflammation, oxidative stress, tissue remodeling, and fibrosis [20,21]. Also, the blockade of ALD actions induced through antihypertensive agents amlodipine or aliskiren, prevents renal oxidative stress by increasing NO-cGMP production in diabetic rats [22]. Additionally, spironolactone (SPL), a MR antagonist, has shown to prevent diabetic renal injury by decreasing oxidative stress, inflammation, apoptosis and fibrosis [23–28].

Based on the evidence above described, we studied the SPL effect to inhibit ALD actions in renal epithelia of diabetic rats, and to explore the mechanism through which ALD may modulate the expression of TJ proteins in GL, PT and DT in early diabetic nephropathy.

## Materials and methods

### Antibodies and chemicals

Rabbit anti-cldn-2, anti-cldn-5 and anti occldn and mouse anti-cldn-4, rabbit anti-cldn-4 and -8, peroxidase-conjugated anti-rabbit, peroxidase- conjugated anti-mouse, Alexa Fluor^®^ 488 donkey anti-rabbit, Alexa Fluor^®^ 488 donkey anti-goat, Alexa Fluor^®^ 594 donkey anti-mouse antibodies, 4',6-diamidino-2-phenylindole (DAPI) and recombinant protein G-Agarose were purchased from Invitrogen (Carlsbad, CA, USA). Goat anti-kidney injury molecule-1 (Kim-1) was purchased from R&D Systems (McKinley Place, MN, USA). Mouse anti-Hsp70/72 antibody was purchased from Enzo Life Sciences, Inc. (Farmingdale, NY, USA). The mouse anti-dipeptidylpeptidase (DppD) antibody was purchased from AbD Serotec (Raleigh, NC, USA). Mouse anti-desmoplakin (DMPK) antibody was purchased from MP Biomedicals (Solon, OH, USA). Mouse anti-phospho-serine, mouse anti-phospho-threonine and rabbit anti-serum and glucocorticoid-induced kinase 1 (SGK1) antibodies were purchased from EMD Millipore (Billerica, MA, USA). Mouse anti-glyceraldehyde 3-phosphate dehydrogenase (GAPDH), goat anti-cldn-8, goat anti-with-no-lysine kinase 4 (WNK4), goat anti-CD4, mouse anti-vascular endothelial (VE)-cadherin, rabbit anti-nephrin and anti-Wilm´s Tumor 1 (WT1) and peroxidase-conjugated anti-goat antibodies were purchased from Santa Cruz Biotech, Inc. (Santa Cruz, CA, USA). Spironolactone (SPL), Percoll, streptozotocin (STZ), glutathione reduced form (GSH), tetramethoxypropane, streptomycin sulfate, 1-methyl-2-phenylindole, butylated hydroxytoluene (BHT), nicotinamide adenine dinucleotide phosphate (NADPH), salmon testes DNA, and diphenyleneiodonium chloride (DPI) were purchased from Sigma-Aldrich Co. (St. Louis, MO, USA).

### Ethical treatment of animals

This study was carried out in strict accordance with the recommendations in the Guide for the Care and Use of Laboratory Animals of the Mexican Official Norm NOM-062-ZOO-1999. The protocol was approved (protocol # 491) by the Committee on the Ethics of Animal Experiments of the Institutional Animal Care Committee (UPEAL). All surgery was performed under sodium pentobarbital anesthesia, and all efforts were made to minimize suffering.

### Experimental design

**Fig 1:**
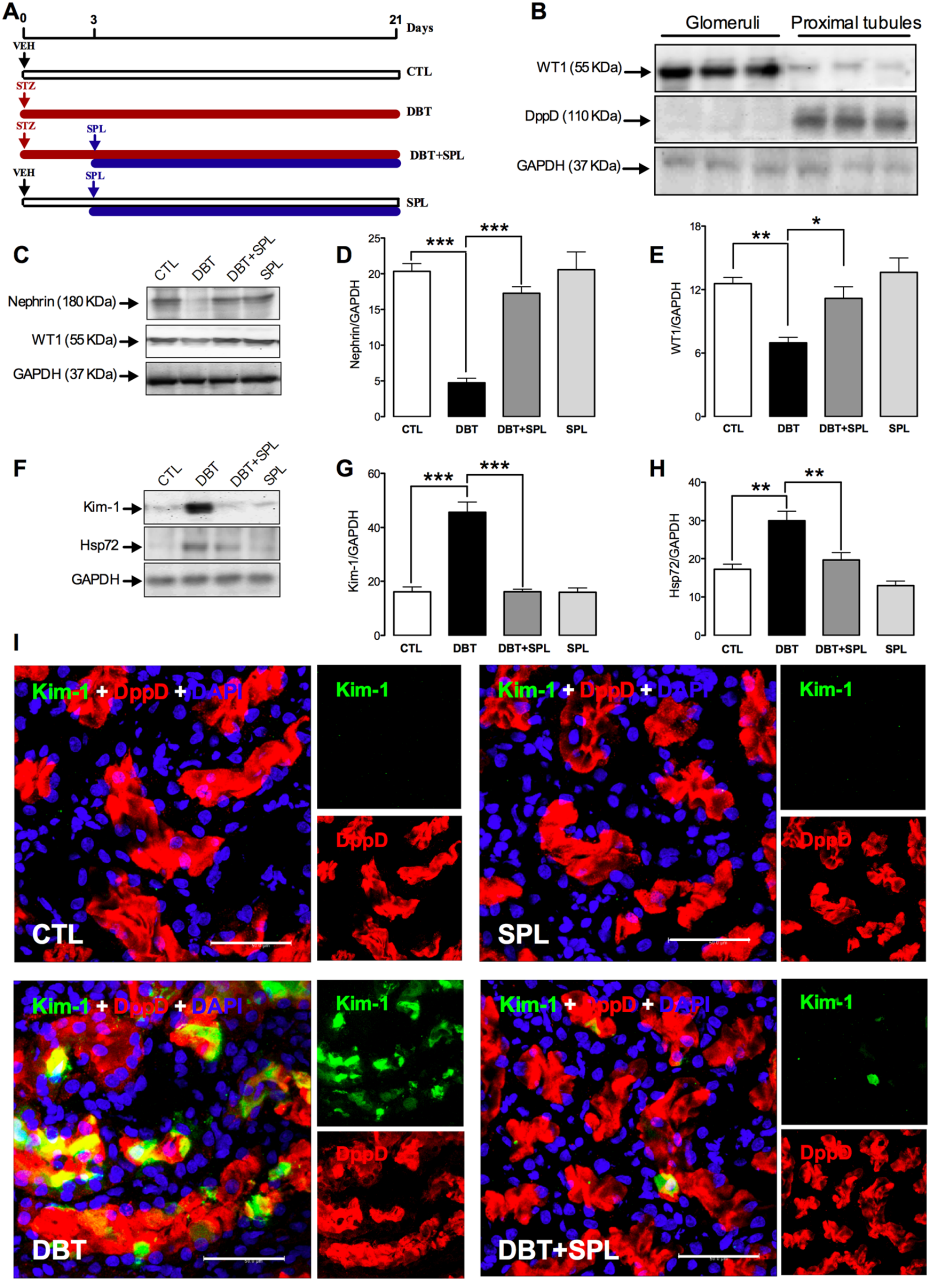
SPL treatment prevents diabetes-induced glomerular and tubular damage. (A) Schematic representation of the experimental strategy used in this study. Four groups of rats were studied: Control (CTL), Diabetic (DBT), DBT+SPL and SPL, respectively (for detail see Material and methods). (B) Purity of isolated glomerular and proximal tubular segments was evaluated by IB analysis of Wilm´s tumor 1 (WT1) and dipeptidylpeptidase (DppD) expressions, respectively. (C) IB analysis of nephrin and WT1 shows that SPL treatment prevents diabetes-induced glomerular damage; densitometric analysis of nephrin (D) and WT1 (E) are shown (S1 Fig). (F) IB analysis of kidney injury molecule 1 (Kim)-1 and heat shock protein of 72 KDa (Hsp72) show that SPL treatment decreases diabetes-induced proximal tubular damage, densitometric analysis of Kim-1 (G) and Hsp72 (H) are shown (S1 Fig). No changes were found in SPL group compared to CTL group. (I) IF analysis confirms that SPL treatment decreases diabetes-induced Kim-1 expression (green label) in PT labeled with DppD (red label). Nuclei were marked with 4', 6-diamidino- 2-phenylindole (DAPI, blue label). No significant changes were found between SPL and CTL groups. Glyceraldehyde 3-phosphate dehydrogenase (GAPDH) was used as loading control for IB. Data are mean±SEM from 3 rats per group. *p<0.05; **p<0.01 and ***p<0.001. Images are representative of three different experiments performed in the four experimental groups. Bar = 50 μm.

Female Wistar rats with an initial body weight of 200–250 g were housed with 12/12 h light/dark cycles at 22±1°C and 50±5% humidity. Animals received water and food ad libitum. Type 1 diabetes was induced by a single tail vein injection of STZ (60 mg/kg body weight) diluted in citrate buffer, pH 6.0. Non-diabetic control rats were injected with an equal volume of citrate buffer. After 3 days, blood glucose was measured with OneTouch^®^ Ultra blood glucose meter (Milpitas, CA, USA) to confirm the induction of diabetes. SPL was dissolved in carboxymethylcellulose (0.2%) and given daily by gavage (20 mg/kg body weight) at a volume 250 μL to DBT+SPL- and SPL-treated groups. Rats were sacrificed 21 days after STZ or vehicle administration. Four groups of rats were studied (n = 12/group): (1) control group, treated daily via oral gavage with carboxymethylcellulose (SPL-vehicle) from days 3–21 after a single injection of citrate buffer (STZ-vehicle); (2) Diabetic (DBT) group, treated daily via oral gavage with carboxymethylcellulose from days 3–21 after STZ administration; (3) DBT+SPL group, SPL was given daily via oral gavage from days 3–21 after STZ injection; and (4) SPL group, SPL was given daily via oral gavage from days 3–21 after injection of citrate buffer (Fig 1A). Rats were kept in metabolic cages from days 20–21 to collect 24 h urine for the measurements of proteinuria, creatinine, Na^+^ and K^+^.

### Biochemical and physiological parameters

Blood glucose and body weight were monitored at days 3, 7, 14 and 21 throughout the study. For blood glucose analysis rats were anaesthetized with sodium pentobarbital (40 mg/kg, i.p.) and blood was collected by the tail vein, for this purpose, collection site was wiped with 70% ethanol prior to blood collection, blood sample was obtained by pricking the vein using a sterile needle, the first drop was wiped with cotton and next was collected for reading with the OneTouch^®^ Ultra blood glucose meter. At day 21, blood was collected by cardiac puncture under anesthesia with sodium pentobarbital (30 mg/kg, i.p.) and serum was separated. Animals were sacrificed by exsanguination under anesthesia as mentioned above and kidneys were excised and weighed. Total urinary protein was measured by Lowry method (Bio-Rad Protein Assay Kit; Bio-Rad Laboratories, Hercules, CA, USA). Creatinine clearance was calculated with the standard formula, and urinary and serum creatinine, were measured by modified Jaffé reaction as previously described. Urinary protein to creatinine ratio was obtained by dividing urine protein concentration by urine creatinine concentration, both expressed in mg/dL. Urinary and serum Na^+^ and K^+^ concentrations were measured by atomic absorption spectrophotometry (Perkin Elmer 3100 with an air- acetylene flame, Norwalk, CT, USA) as previously described [29]. Na^+^ and K^+^ fractional excretion were calculated according to the following equation: Fractional excretion of Na^+^ or K^+^ (%) = (Na^+^ or K^+^ clearance/creatinine clearance) x 100. Serum ALD was measured by using the Human Aldosterone ELISA kit (BioVendor, Asheville, NC, USA) by following the manufacturer´s instructions. Plasma osmolality was measured by a vapor pressure method (Wescor 5500, Portland, OR).

### Extraction of renal tissues, isolation of GL, PT and DT, and protein extraction

GL were isolated as previously described [9]. Briefly, capsules were removed and kidney cortex was separated from the medulla and minced on a glass dish. The homogenized tissue was pushed through a stainless sieve with a pore size of 117 mm (Cat. No. 8321A44; Thomas Scientific, Swedesboro, NJ, USA) applying gentle pressure with the bottom of a glass flask. The sieve was rinsed several times with potassium phosphate-buffered solution (PBS). The sieved tissue containing a preparation enriched in GL was transferred to a second sieve with a pore opening of 74 mm (Cat. no. 8321A58; Thomas Scientific). After several washes with cold PBS, the material that remained on top of the sieve, which contained the GL, was collected in ice-cold PBS and centrifuged for 10 min at 20,000 x g. The supernatant was decanted and the pellet containing the glomeruli was suspended in PBS.

To isolate PT and DT, kidneys from the four experimental groups were perfused with isotonic saline solution (0.9% NaCl), excised, and placed on ice-cold saline solution. Kidneys were decapsulated, and the cortex was removed. Renal tubules were isolated by Percoll gradients as previously described [9,30]. Briefly, renal cortex was minced and then placed in 15 ml of ice-cold Krebs-Bicarbonate solution (KB): 110 mM NaCl, 25 mM NaHCO_3_, 3 mM KCl, 1.2 mM CaCl_2_, 0.7 mM MgSO_4_, 2 mM KH_2_PO_4_, 10 mM sodium acetate, 5.5 mM glucose, 5 mM alanine, and 0.5 g/L bovine serum albumin (BSA), pH 7.4, osmolarity 290 mOsm/kg H_2_O, washed thrice, and resuspended in 10 ml of KB containing 15 mg of type-II collagenase and 0.5 ml of 10% BSA. Samples were gassed with 95% air/5% CO_2_ in a shaking water bath for 20 min at 37°C. After digestion, approximately 20 ml of ice-cold KB solution with a protease inhibitor cocktail complete 1X (Boehringer Mannheim, Germany) and PMSF (1 mM) were added, and the suspension was gently agitated to disperse tissue fragments. Suspension was filtered to remove collagen fibers. Then, tissue suspension was gently centrifuged (18 x g for 30 s). Pellet was resuspended in 10 ml of ice-cold KB with the protease inhibitor cocktail. This washing procedure was repeated thrice. The tissue pellet was then resuspended in 5% BSA with protease inhibitors for 5 min, at 4°C and centrifuged for 1 min, and supernatant was discarded. Tissue pellets were suspended in 30 ml of a freshly prepared mixture of ice-cold Percoll and KB (1:1, v/v). Thereafter, the suspension was centrifuged (1,071 × g for 30 min), resulting in separation of four bands, the first was enriched with DT and the fourth with PT. Band content was confirmed by light microscopy observation as previously described [9]. For total protein extraction, PT and DT suspensions were gently centrifuged (18 × g for 30 s). Pellets were suspended in 10 ml of ice-cold KB containing protease inhibitor cocktail. This washing procedure was repeated thrice. Tissue pellets were suspended and incubated for 30 min at 4°C in 300 μL of lysis buffer (RIPA): 40 mM Tris-HCl pH 7.6, 150 mM NaCl, 2 mM EDTA, 10% glycerol, 1% Triton X-100, 0.5% sodium deoxycholate, 0.2% sodium dodecyl sulfate (SDS), 1 mM sodium orthovanadate, 0.5 mM sodium fluoride, 1 mM PMSF and complete 1X. Thereafter, samples were sonicated thrice for 30 s each in a high-intensity ultrasonic processor (Vibra cell; Sonics & Materials Inc., Danbury, CT, USA), centrifuged at 20,000 x g, at 4°C, for 40 min and supernatants were collected.

### Immunoblot (IB) analysis

IB was performed as previously described [9]. Briefly, samples were diluted 1:5 in 5x Laemli buffer with 20 mM urea, and then denatured by boiling for 12 min. Proteins were loaded on SDS-PAGE 12% gels. Molecular weight standards (Amersham Pharmacia Biotech, Piscataway, NJ, USA) were run in parallel. Proteins were transferred to polyvinylidene fluoride (PVDF) membranes (Amersham Biosci, Uppsala, Sweden). Nonspecific protein binding was blocked by incubation with 1X casein solution (Vector Laboratories, Inc. Burlingame, CA, USA), for 1 h, at room temperature. Membranes were incubated overnight at 4°C with the appropriate primary antibodies against GAPDH, cldn-2, -4, -5, and -8, occldn, WT1, DppD, nephrin, Kim-1, Hsp72, WNK4, phospho-serine, phospho-threonine, and SGK1 (dilution 1:500). Thereafter, membranes were incubated with peroxidase-conjugated anti-rabbit, anti-mouse or anti-goat (dilution 1:10,000) antibodies for 1 h, after washing, immunoblots were developed using the ECL^™^ prime Western blotting detection reagent (Amersham^™^, GE Healthcare, Buckinghamshire, UK). Chemiluminescence was detected in an EC3 Imaging System (UVP Biolmaging Systems, Cambridge, UK). Protein band density was quantified by transmittance densitometry (UVP BioImaging Systems software, Cambridge, UK).

### Immunofluorescence (IF), immunocytochemistry and confocal microscopy analysis

Kidney samples were prepared for IF as previously described [9,31]. Briefly, cubes of 0.5 cm/side were cut and immediately immersed for 5 min in 2-methylbutane (Aldrich M3, 263–1; Milwaukee, WI, USA), which was immediately cooled in liquid nitrogen. Next, 6 μm sections were cut in a Leica CM 1510 cryostat (Wetzlar, Germany) and mounted on gelatin-coated slides that were then kept frozen at -70°C. For the IF experiments, the sections were fixed for 10 min with methanol and subsequently incubated for 5 min at room temperature in 0.5% (vol/vol) Triton X-100. Then, the tissue sections were washed thrice for 5 min with PBS, blocked for 1 h at room temperature with 0.5% (wt/vol) IgG-free albumin (1331-A, Research Organics, Cleveland, OH, USA), and incubated overnight at 4°C with one of the following primary polyclonal antibodies: rabbit anti-cldn-2, -4, -5, and -8, and rabbit anti-occldn, goat anti-Kim-1 and anti-WNK4 (dilution 1:100) and mouse anti-DMPK (dilution 1:50) and anti-DppD (1:300). DppD and DMPK antibodies were used to identify PT and DT, respectively. Secondary antibodies Alexa Fluor^®^ 488 donkey anti-rabbit, Alexa Fluor^®^ 488 donkey anti-goat, Alexa Fluor^®^ 594 donkey anti-mouse (dilution 1:500) and DAPI (to label nuclei) were used. IF was evaluated using a confocal inverted microscope (TCS SP8, Leica, Heidelberg, Germany). IF experiments were performed at least thrice in samples from three different animals per group. Nonspecific labeling was assessed by exclusion of the primary antibodies and by using a non-specific IgG.

### RNA extraction, cDNA synthesis and quantitative real-time polymerase chain reaction (qRT-PCR)

Total RNA was extracted from renal cortex using the Trizol reagent (Invitrogen) according to the manufacturer’s instructions. Concentration and quality of the RNA were measured using a Genesys 10uv spectrophotometer (Thermo Scientific, Hudson, NH, USA) and the integrity was determined by gel electrophoresis. cDNA synthesis was carried out under the following conditions: 5 μg of total RNA was mixed with 1X First-Strand buffer (250 mM Tris-HCl pH 8.3, 375 mM KCl, 15 mM MgCl_2_), 10 mM DTT, and 0.5 mM of each deoxynucleotide triphosphate, 150 ng random primers and 200 U of M-MLV reverse transcriptase (Invitrogen) and incubated for 50 min at 37°C followed by inactivation at 70°C for 15 min. RT-PCR was done using the SDS 7500 system (Applied Biosystems, Foster City, CA, USA) with SYBR Green detection. Reaction was carried out in a 12.5 μL final reaction volume containing 7.5 μL of 2X Maxima SYBR Green/ROX qRT-PCR Master Mix (Fermentas) 0.5 μM each forward and reverse primer and 5.0 μL cDNA solutions. Thermal profile for the RT-PCR was 95°C for 10 min, followed by 40 cycles of 95°C for 15 s, 60°C for 60 s. Expression of individual genes was normalized against the expression of GAPDH gene, and levels were measured by comparative Ct method. Primer sequences (forward, reverse) and lengths of the amplified products were as follows: cldn-2 (5´-TTCATCCTTGGTGGTATC-3´, 5´-CAGTGGTGAGTAGAAGTC-3´; 81), cldn-4 (5´-GCCAGCAACTATGTGTAAG-3´, 5´-GCCGTTATGAGTTCAATCC-3´; 75), cldn-5 (5´-GTCCAGAGTTCAGTTTTC-3´, 5´-TAGTTCTTCTTGTCGTAATC-3´; 79), cldn-8 (5´-CCGAGCATATACTCCAAA-3´, 5´-GTACGAGGCAGTTAAGAA-3´; 154), occldn (5´-TTGCTTCATCGCTTCCTT-3´, 5´-TCAAGTAGTATCTTCTCGTTCTG-3´; 76), WNK4 (5´-TAGACTGGCACCCATATC-3´, 5´-TGGTTCCTTGGATGAAGT-3´; 76), and GAPDH (5´-CTTGGGCTACACTGAGGACC-3´, 5´-CTGTTGCTGTAGCCGTATTC-3´; 100).

### Oxidative stress markers

#### O_2_^●―^ production assay

Fluorescence detection of O_2_^●―^ production in GL and PT was performed as described by Trujillo et al. (2015) [32]. Briefly, fluorescence was detected by conversion of DHE to ethidium (Eth). Isolated GL and PT were homogenized in 500 μL of a phosphate-buffered saline (PBS) solution. Homogenates were subjected to a low-speed centrifugation (800 × g/10 min, 4°C) to remove the unbroken cells and debris, and aliquots were used immediately. GL and PT homogenates (20 μg) were incubated with DHE (0.02 mmol/L), salmon testes deoxyribonucleic acid (DNA) (0.5 mg/mL) and NADPH (1.125 mM) as substrate or diphenyleneiodonium (DPI) as inhibitor (1.125 mM). The assay was performed in a microtiter plate placed away from direct light at 37°C for 30 min. Eth-DNA fluorescence was measured at an excitation of 480 nm and an emission of 610 nm by using the Synergy HT multimode microplate reader (Biotek Instruments, Winooski, VT, USA). The fluorescence intensity of each sample was normalized relative to the control. The protein content was measured using the Lowry method.

#### Lipid peroxidation

Lipid peroxidation was assessed in isolated GL and PT by measuring MDA and 4-hydroxynonenal (4-HNE) using a standard curve of tetramethoxypropane as previously described [9]. Data are expressed as nmol MDA + 4-HNE/mg protein.

#### Protein carbonylation

For protein carbonyl content isolated GL and PT samples were incubated overnight with streptomycin sulfate to remove nucleic acids. Later, homogenates were treated with dinitrophenylhydrazine (DNPH) and HCl and finally with guanidine hydrochloride. Assessment of carbonyl formation was obtained on the basis of formation of protein hydrazone by reaction with DNPH. The absorbance was measured at 370 nm. Protein carbonyl content is expressed as nmol/mg protein as previously described [9].

#### GSH content

GSH levels were measured in isolated GL and PT by using monochlorobimane as previously described [9]. The fluorescence was measured at excitation and emission wavelengths 385 and 478 nm, respectively, using a Synergy HT multimode microplate reader. GSH levels are expressed as μmol/mg protein.

### Immunoprecipitation (IP)

IP of cldn-4, -8 and WNK4 were performed in isolated DT with mouse anti-cldn-4 and -8 and goat anti-WNK4 (10 μg) as previously described [9]. Briefly, fresh DT extracts (1 mg) were precleared with 20 μl of recombinant protein G-agarose beads overnight at 4°C. The beads were removed by centrifugation at 16,000 x g for 5 min, and precleared extracts were incubated overnight at 4°C with 2.5 mg of the immunoprecipitating antibody previously bound to protein G-agarose. As negative control, parallel incubations with an irrelevant antibody (mouse anti-VE-cadherin (10 μg) for cldn-4 and -8 and goat anti-CD4 (10 μg) for WNK4) were performed. Immune complexes were collected by centrifugation at 16,000 x g for 5 min and washed thrice with 1 ml of RIPA buffer and then, eluted by boiling in 300 μL of RIPA buffer. Immunoprecipitated proteins were then analyzed by IB as described above. GAPDH was analyzed in input extracts as loading control.

### Data analysis

Results are expressed as mean±standard error of the mean (SEM). One-way analysis of variance (ANOVA) was used for multiple comparisons among groups. Bonferroni post hoc test was performed, and p<0.05 was considered statistically significant.

## Results

### SPL partially prevents body weight loss but not hyperglycemia in diabetic rats

To inhibit the ALD-induced MR activation, rats were treated with SPL, whose administration has shown to exert nephroprotection in diabetic renal injury [23–28]. Our first aim was to determine the effect of SPL on blood glucose in diabetic rats. As shown in Table 1, DBT+SPL group showed similar blood glucose levels compared to DBT group. Also, SPL treatment partially prevents diabetes-induced loss of body weight, and SPL group showed similar body weight and blood glucose concentration as compared to control group (Table 1). These findings indicate that SPL partially prevented body weight loss without effect on high blood glucose levels induced by streptozotocin (STZ) administration. Additionally, SPL significantly decreased diabetes-induced increment in kidney weight and kidney/body weight ratio (Table 1), thus suggesting a nephroprotective role of SPL.

**Table 1:** Physiological and biochemical parameters of rats.

Parameters	CTL	DBT	DBT+SPL	SPL
Blood glucose (mg/dL)	95.4 ±3.3	451.2 ±16.2***	459.6 ±21.1***	96.0 ±6.9
Body weight (g)	293 ±5.5	215 ±2.8***	261 ±4.7^##^	291 ±15.7
Kidney weight (g)	0.83 ±0.04	1.16 ±0.04***	0.96 ±0.05^##^	0.82 ±0.03
Kidney/body weight ratio	0.0028 ±0.0007	0.0054 ±0.0004**	0.0036 ±0.0003^#^	0.0028 ±0.0001
Serum aldosterone (pg/mL)	589 ±22	700 ±29*	751 ±39*	592 ±33
Proteinuria (mg/day)	5.9 ±0.9	15.4 ±1.6*	8.9 ±0.7^#^	5.9 ±1.1
Proteinuria/creatininuria ratio	0.61 ±0.03	1.34 ±0.18*	0.76 ±0.03^#^	0.68 ±0.09
Serum creatinine (mg/dL)	1.20 ±0.06	1.35 ±0.07	1.25 ±0.09	1.28 ±0.04
Creatinine clearance (mL/min)	1.11 ±0.08	1.19 ±0.13	1.04 ±0.10	1.17 ±0.11
Serum Na^+^ (mEq/L)	141 ±2.2	143 ±2.0	139 ±2.9	138 ±1.1
Urinary Na^+^ (mEq/day)	12.4 ±1.8	28.0 ±4.3*	15.9 ±3.1^#^	14.6 ±2.3
FENa^+^ (%)	0.30 ±0.02	0.65 ±0.06**	0.33 ±0.05^#^	0.36 ±0.07
Serum K^+^ (mEq/L)	5.1 ±0.7	5.3 ±0.8	5.2 ±1.1	5.1 ±0.6
FEK^+^ (%)	31.4 ±2.6	28.6 ±2.8	28.2 ±3.1	32.1 ±4.3
Plasma osmolality (mg/kg H_2_O)	296 ±1.9	321 ±11.3*	322 ±8.4*	305 ±4.2

CTL, control group; DBT, diabetic group; DBT+SPL, diabetes + spironolactone group; SPL, spironolactone group; FENa^+^, fractional excretion of sodium; FEK^+^, fractional excretion of potassium. Values are represented as means ± SEM (n = 6–8). *p<0.05; **p<0.01 and ***p<0.001 vs control and #p<0.05; ##p<0.01 vs DBT.

### SPL does not have effect on serum K^+^ and ALD levels but prevents diabetes-induced proteinuria and natriuresis

Next set of experiments were designed to analyze the effect of SPL on diabetes-induced renal dysfunction. As shown in Table 1, SPL significantly decreased proteinuria and proteinuria/creatininuria ratio compared to DBT group. SPL group did not show differences compared to control group. Furthermore, serum creatinine, creatinine clearance, FEK^+^ and serum Na^+^ and K^+^ did not change in the four experimental groups studied. It was important to measure serum K^+^ level due to MR blockade is a major risk for hyperkalemia [33]. However, no changes were found in the levels and FEK^+^ (Table 1), indicating that the dose of SPL used in this study was safe for the rats. Additionally, under diabetic conditions, SPL treatment ameliorated diabetes-induced increased urinary Na^+^ and FENa^+^, and did not have effect on plasma osmolality (Table 1). On the other hand, serum ALD levels were analyzed in the four experimental groups, and it was found that 3 weeks after diabetes induction, serum ALD increased significantly compared to control group. However, DBT+SPL group showed similar serum ALD levels compared to DBT group (Table 1). The data described above suggests that the increased plasma osmolality may be a consequence of increased blood glucose concentration and dehydration, which may lead to increased synthesis of ALD. The findings related to decreased proteinuria, natriuresis and FENa^+^ suggest that SPL provides nephroprotective effects in GL and PT.

### SPL prevents diabetes-induced glomerular and proximal tubular damage

We have previously reported that diabetes induces renal damage mainly in GL and PT, and in a lesser extent in DT [9]. Next set of experiments were conducted to explore the effect of SPL on diabetes-induced renal damage. In order to show the purity of glomerular and proximal tubular isolated sections, IB analyzes of WT1 and DppD were used as markers of glomerular and proximal tubular segments (Fig 1B). As shown, DppD and WT1 expressions are absent in glomerular and proximal tubular fractions, respectively. These data confirm the purity of the isolated nephron segments. To analyze glomerular injury, nephrin and WT1 were used as markers of damage, it was found that SPL ameliorated diabetes-induced decreased expressions of nephrin (Fig 1C and 1D) and WT1 (Fig 1C and 1E). Also, the expressions of Kim-1 and Hsp72 were analyzed as markers of proximal tubular damage. It was found that SPL prevented diabetes-induced increased Kim-1 (Fig 1F and 1G) and Hsp72 (Fig 1F and 1H) expressions, thus suggesting a nephroprotective role of SPL. It is important to mention that similar results were obtained between control (CTL) and SPL groups. Additionally, cellular localization of Kim-1 was explored by IF, it was found that SPL decreased diabetes-induced renal expression of Kim-1 (Fig 1I, green label) in proximal tubules marked with DppD (red label). No differences were observed between CTL and SPL groups. In conclusion, the data above described demonstrate that SPL administration prevents diabetes-induced glomerular and proximal tubular damage that may be associated to decreased proteinuria, natriuresis and FENa^+^.

### SPL prevents diabetes-induced decreased protein expression of cldn-5 in GL, and cldn-2 and occldn in PT

**Fig 2:**
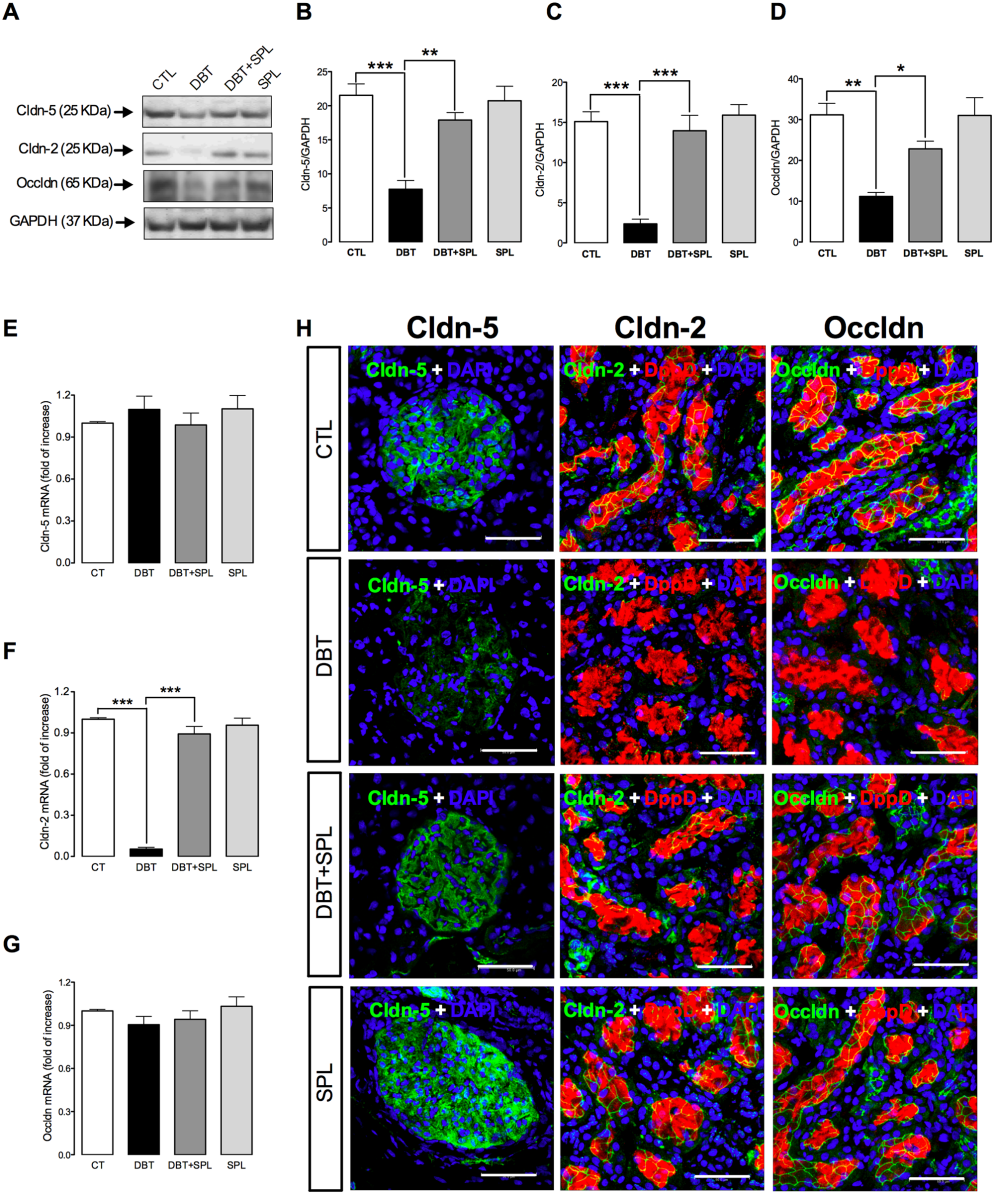
SPL treatment decreases diabetes-induced decrement in protein expression of cldn-5 in glomeruli (GL) and cldn-2 and occldn in proximal tubules (PT). SPL prevents diabetes-induced decrease of cldn-5 protein expression (A and B) in GL, and mRNA (F) and protein expression of cldn-2 (A and C), and protein expression of occldn (A and D) in PT (S2 Fig). However, diabetes did not have effect in the mRNA levels of cldn-5 (E) and occldn (G) evaluated by qRT-PCR (S2 Fig). Additionally, cellular localization of cldn-2 and -5 and occldn were assessed by IF (H). SPL-treatment prevents diabetes-induced loss of cldn-5 (green label) in the cell borders of GL and, cldn-2 (green label) and occldn (green label) in the cell borders of PT labeled with DppD (red label), nuclei were marked with DAPI (blue label). No changes were found between CTL and SPL groups. GAPDH was used as loading control. Densitometric analysis of IBs from the four experimental groups are shown in panels B for cldn-5, C for cldn-2 and D for occldn. Data are mean±SEM from 3 rats per group. *p<0.05; **p<0.01 and ***p<0.001. Images are representative of three different experiments performed in the four experimental groups. Bar = 50 μm.

Next experiments were performed to explore the effect of SPL in diabetes-induced loss of renal TJ proteins in GL and PT. In order to explore this issue, cldn-5, which is abundantly expressed in glomerular endothelia and podocytes [6,8], and whose decreased expression is associated to increased proteinuria [9,34], was analyzed. Also, expressions of occldn and cldn-2 were analyzed in isolated PT. It was found that SPL treatment prevented diabetes-induced loss of cldn-5 (Fig 2A and 2B) in GL, and cldn-2 (Fig 2A and 2C) and occldn (Fig 2A and 2D) in PT evaluated by IB. The data above described were confirmed by IF assays (Fig 2H). It is observed that in CTL conditions cldn-5 (green label) is distributed in all the glomerular structure, in contrast under diabetic condition, this immunofluorescence signal is less intense and discontinuous. SPL treatment prevented diabetes-induced loss of cldn-5 (Fig 2H). On the other hand, in PT marked with DppD (red label) the signal of cldn-2 (green) and occldn (green) showed a characteristic chicken fence pattern which almost disappeared under diabetic condition. However, SPL treatment decreased diabetes-induced loss of both proteins in PT. Additionally, we explore the mRNA levels of these proteins, it was found that diabetes did not change cldn-5 (Fig 2E) and occldn (Fig 2G) expressions, thus suggesting that loss of both proteins is consequence of post translational modifications. In contrast, it was found that diabetes decreased mRNA levels of cldn-2 (Fig 2F) and that SPL ameliorated this change. These results suggest that the decreased protein of cldn-2 is associated with its low mRNA levels.

### SPL prevents diabetes-induced oxidative stress in GL and PT

**Fig 3:**
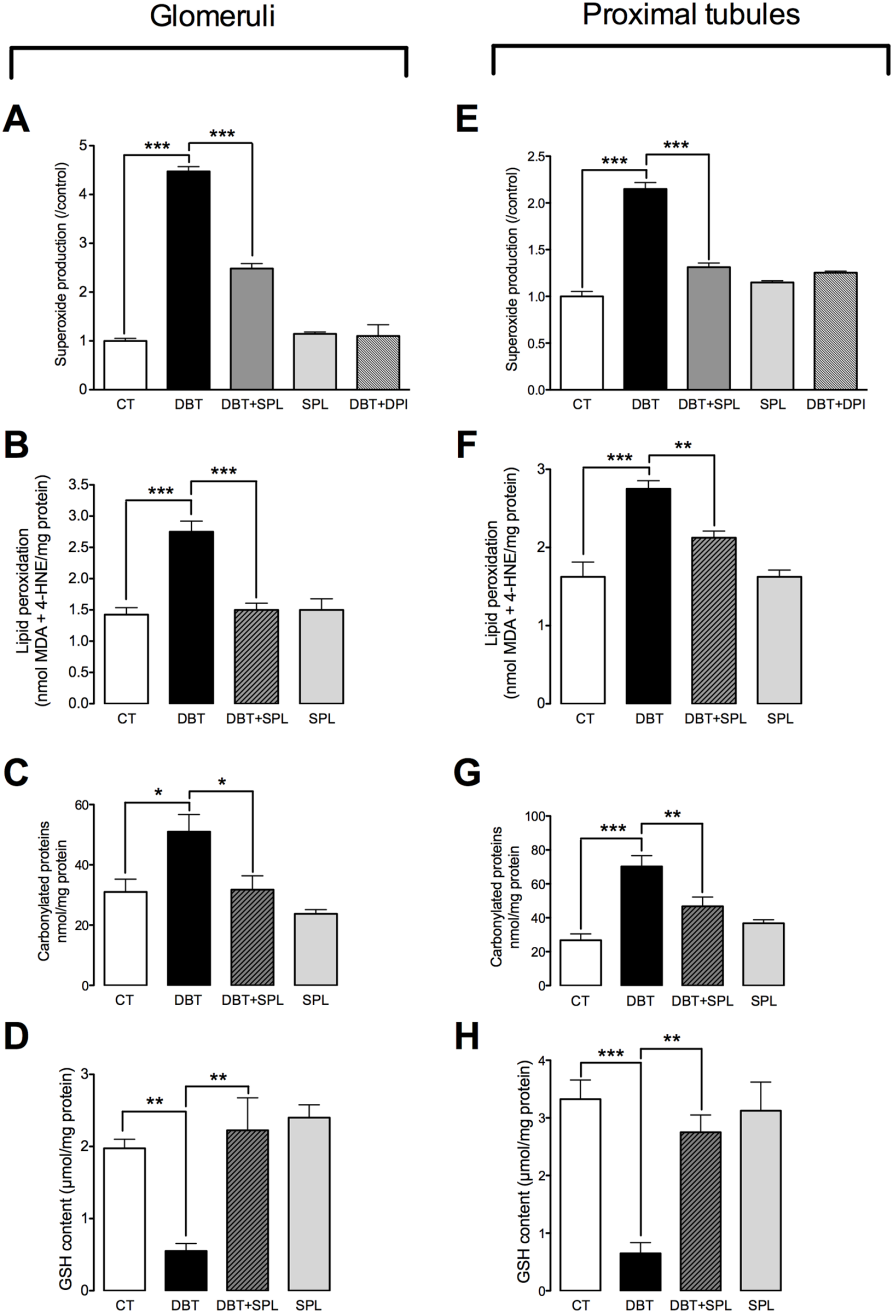
SPL decreases diabetes-induced oxidative stress in GL and PT. To evaluate oxidative stress, superoxide anion (O_2_^●―^) production, lipid peroxidation, protein carbonylation and reduced glutathione (GSH) content were measured in isolated GL and PT. SPL treatment prevents diabetes-induced increment in O_2_^●―^ production (A) by using nicotinamide adenine dinucleotide phosphate (NADPH) as substrate and, diphenyleneiodonium (DPI) as inhibitor, lipid peroxidation (B) and protein carbonylation (C) and decreased GSH content (D) in GL (S3 Fig). Also, SPL diminished diabetes-induced increment of O_2_^●―^ production (E), lipid peroxidation (F) and protein carbonylation (G) and decreased GSH content (H) in PT (S3 Fig). Similar results were found between CTL and SPL groups. Data are mean±SEM from 5–6 rats per group. *p<0.05; **p<0.01 and ***p<0.001.

It has been previously described that high ALD levels may induce oxidative stress in tubular epithelial cells and glomeruli [35,36]. Also, ALD induces glomerular podocyte injury, causing the disruption of the glomerular filtration barrier and proteinuria by increasing oxidative stress [37]. In order to explore whether SPL administration attenuates early diabetes-induced oxidative stress, O_2_^●―^ production, lipid peroxidation, carbonylated proteins and GSH content were measured in isolated GL and PT. It was found that SPL prevented diabetes-induced increased O_2_^●―^ production in GL (Fig 3A) and PT (Fig 3E) by using NADPH as substrate. Also, DPI, a NADPH oxidase inhibitor, decreased O_2_^●―^ production similarly as SPL (Fig 3A and 3E) in both nephron segments (GL and PT). These data are consistent with previously reported, that early diabetes-induced O_2_^●―^ production was mediated by NADPH oxidase activation [9]. Additionally, SPL prevented diabetes-induced increased lipid peroxidation (Fig 3B and 3F) and protein carbonylation (Fig 3C and 3G) and decreased GSH levels (Fig 3D and 3H) in GL and PT, thus confirming the increased oxidative status in both nephron segments and beneficial effect of SPL. The findings above described confirm that diabetes-induced loss of cldn-5 in GL, and cldn-2 and occldn in PT are related to increased oxidative stress, as previously reported [9].

### SPL decreases diabetes-induced overexpression of cldns-4 and -8 in DT

**Fig 4:**
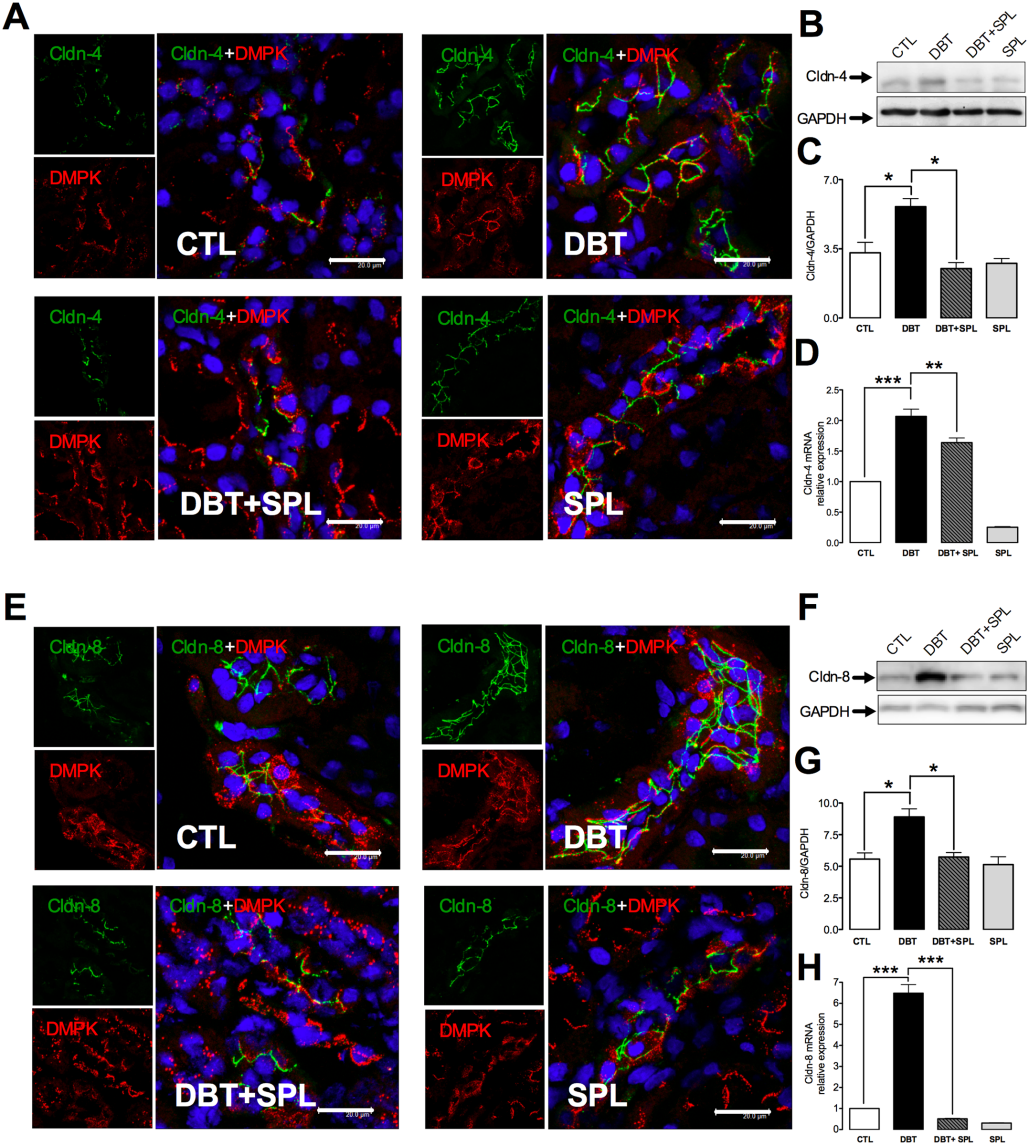
SPL treatment blunts diabetes-induced increment of protein and mRNA expressions of cldn-4 and -8. SPL decreased diabetes-induced cldn-4 and -8 expressions evaluated by IF, IB and qRT- PCR. IF analysis shows that 3 weeks after diabetes induction, increased expressions of (A) cldn-4 (green label) and (E) cldn-8 (green label) were found in the TJs of DT labeled with desmoplakin (DMPK, red label). Nuclei were marked with DAPI (blue label). SPL treatment significantly decreased these changes. To confirm IF findings, IB and qRT-PCR analyzes were performed (S4 Fig). Diabetic condition significantly increased protein (B) and mRNA (D) levels of cldn-4; these changes were decreased by SPL treatment. Also, SPL treatment decreased diabetes-induced increased protein (F) and mRNA (H) levels of cldn-8. Similar results were found between CTL and SPL groups. GAPDH was used as loading control. Densitometric analyzes of IB from the four experimental groups are shown in panels C for cldn-4 and, G for cldn-8 (S4 Fig). Data are mean±SEM from 3 rats per group. *p<0.05; **p<0.01 and ***p<0.001. Images are representative of three different experiments performed in the four experimental groups. Bar = 20 μm.

We previously reported that diabetes does not induce oxidative stress in DT, which partially explains the increased expression of cldn-4 and -8 in this segment [9]. However, it has been reported that ALD induces the cldn-8 transcription in distal colon [38]. To verify, if the ALD regulates cldn-4 and -8 over expression in DT, next set of experiments were conducted to explore this possibility. We evaluated whether SPL treatment prevented cldn-4 and -8 over expressions induced by diabetes. It was found that SPL treatment decreased diabetes-induced cldn-4 and -8 expressions in DT, evaluated by IF (Fig 4A and 4E), IB (Fig 4B and 4F) and qRT-PCR (Fig 4D and 4H). As observed by IF, cldn-4 and -8 are increased in the cell borders (green label) of DT (marked with DMPK, red label) under diabetic condition, and SPL treatment prevented these changes. IBs were performed in the total fraction of isolated DT; it was found that diabetes-induced increment in protein expression was blocked by SPL treatment, thus reinforcing the findings of IF. Densitometric analysis of IB is shown in Fig 4C and 4G. Also, qRT-PCR assays were performed, and it was found that diabetes induced mRNA expression of cldns-4 and -8, and SPL treatment decreased these changes. The findings above described suggest that MR activation by ALD mediates cldns-4 and -8 over-expressions, and their localization in the TJ.

### SPL prevents diabetes-induced increment in co-localization of cldn-4 and -8 in the TJ

**Fig 5:**
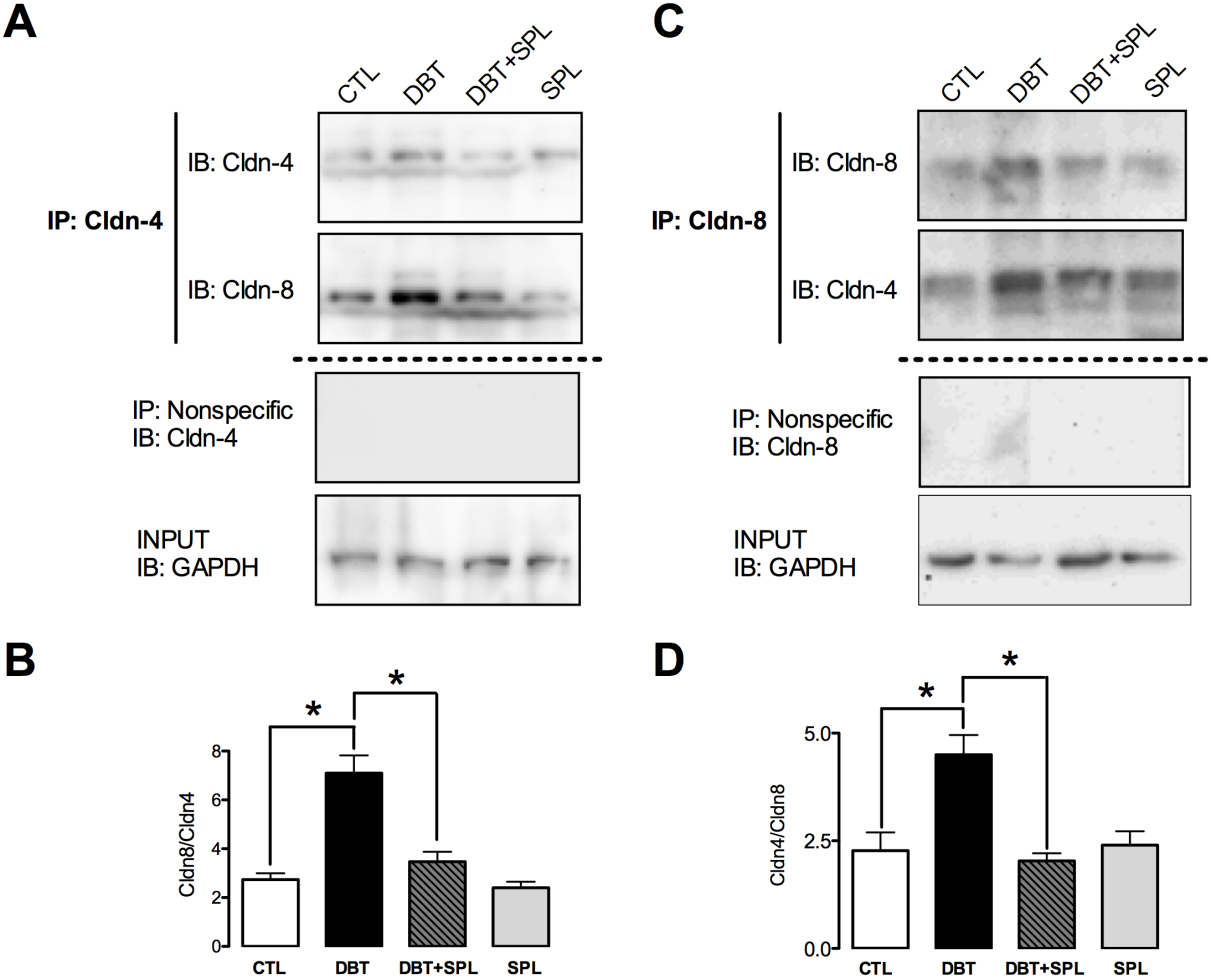
SPL treatment decreases diabetes-induced co-immunoprecipitation (co-IP) of cldn-4 and -8. To evaluate direct interaction of cldn-4 and -8, co-IP analyzes from isolated DT from the four experimental groups were performed. As shown in panel A, cldn-4 co-immunoprecipitated with cldn-8; densitometric analysis (B) shows that diabetic condition increased their interaction and SPL treatment prevents this change (S5 Fig). Also, increased co-IP of cldn-8 with cldn-4 was found under diabetic condition and SPL decreased this interaction (C), densitometric analysis show that these changes were significant (D) (S5 Fig). As shown in panels A and C, no signal was found under nonspecific conditions of IP performed with an unrelated antibody. GAPDH was used in input extract as loading control. Data are mean±SEM from 3 rats per group. *p<0.05.

It has been proposed that in the TJ of tight epithelia, cldns-4 and -8 co-localize, and cis-interact to form a paracellular Cl^―^ channel [16]. Cldn-8-mediated sealing of the paracellular barrier prevents back-leakage of absorbed Na^+^ by ENaC in distal colon [38]. In order to evaluate whether diabetes induces cldn-4 and -8 interactions in the TJ, co-immunoprecipitation (co-IP) analyzes were performed in isolated DT. Increased co-IP of cldn-4 and -8 was found under diabetic condition (Fig 5A–5D), and SPL treatment decreased these changes. Cldn-4 was immunoprecipitated and IB revealed its interaction with cldn-8 (Fig 5A). Also, the results were confirmed by reverse immunoprecipitation (IP) of cldn-8 and IB of cldn-4 (Fig 5C). Densitometric analysis shows that SPL treatment significantly decreased cldn-4 and -8 interactions (Fig 5B and 5D).

### Diabetes induces the expression, co-localization and co-IP of WNK4 with cldn-4 and -8 in the TJs of DT

**Fig 6:**
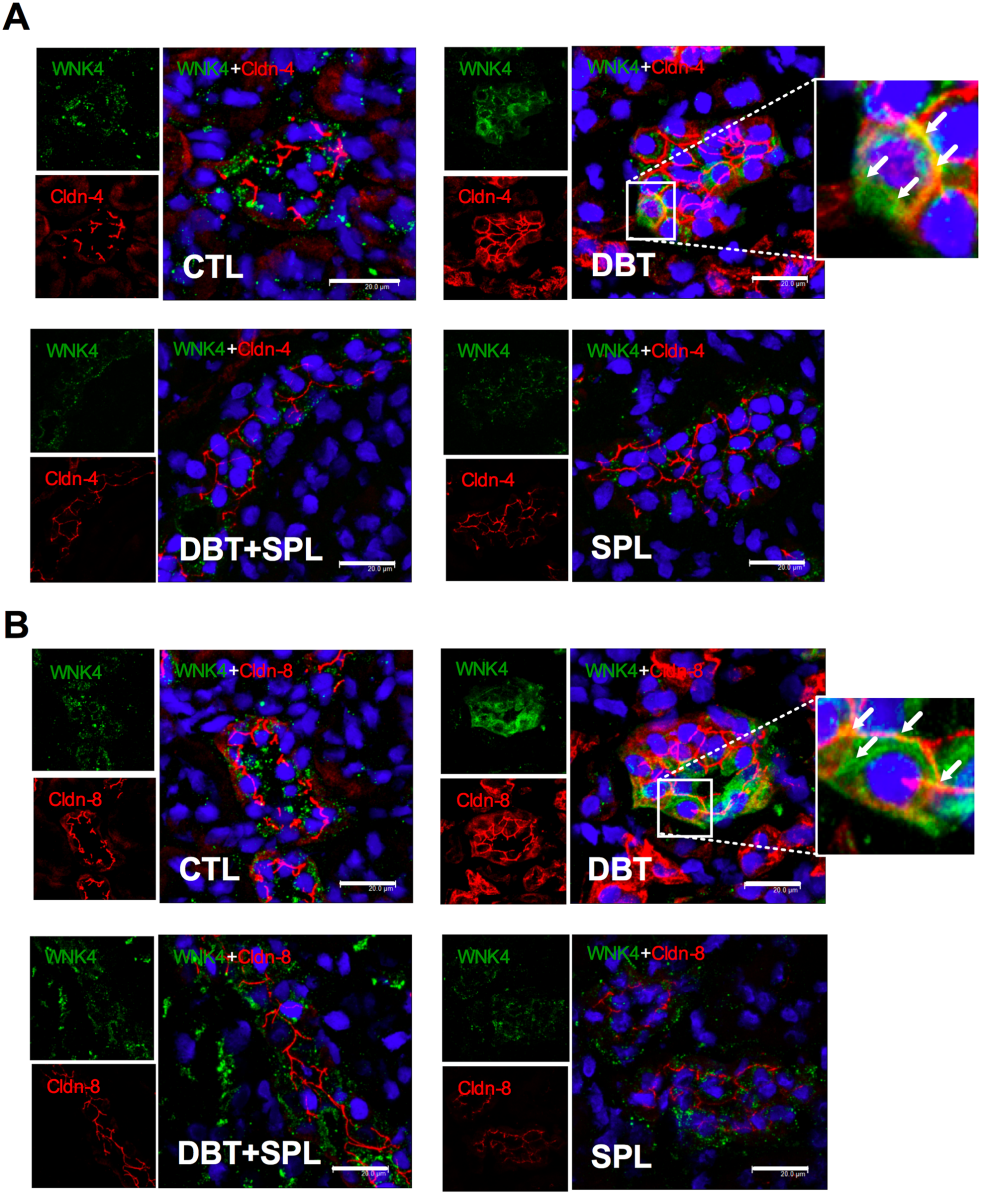
Diabetic condition induces co-localization of WNK4 with cldn-4 and -8 in the TJ of DT. Localization of WNK4 and cldn-4 and -8 was evaluated by IF in the four experimental groups. As shown, diabetes induced co-localization of WNK4 (green label) with cldn-4 (panel A, red label) and, with cldn-8 (panel B, red label) in cell borders of DT. Nuclei were marked DAPI blue label). An approach of WNK4 with cldn-4 and -8 is shown under diabetic condition, showing that WNK4 localizes in cell borders and cytoplasm. SPL treatment decreased co-localization of WNK4 with cldn-4 and -8 induced by diabetes. Similar results were found between CTL and SPL groups. Images are representative of three different experiments performed in the four experimental groups. Bar = 20 μm.

**Fig 7:**
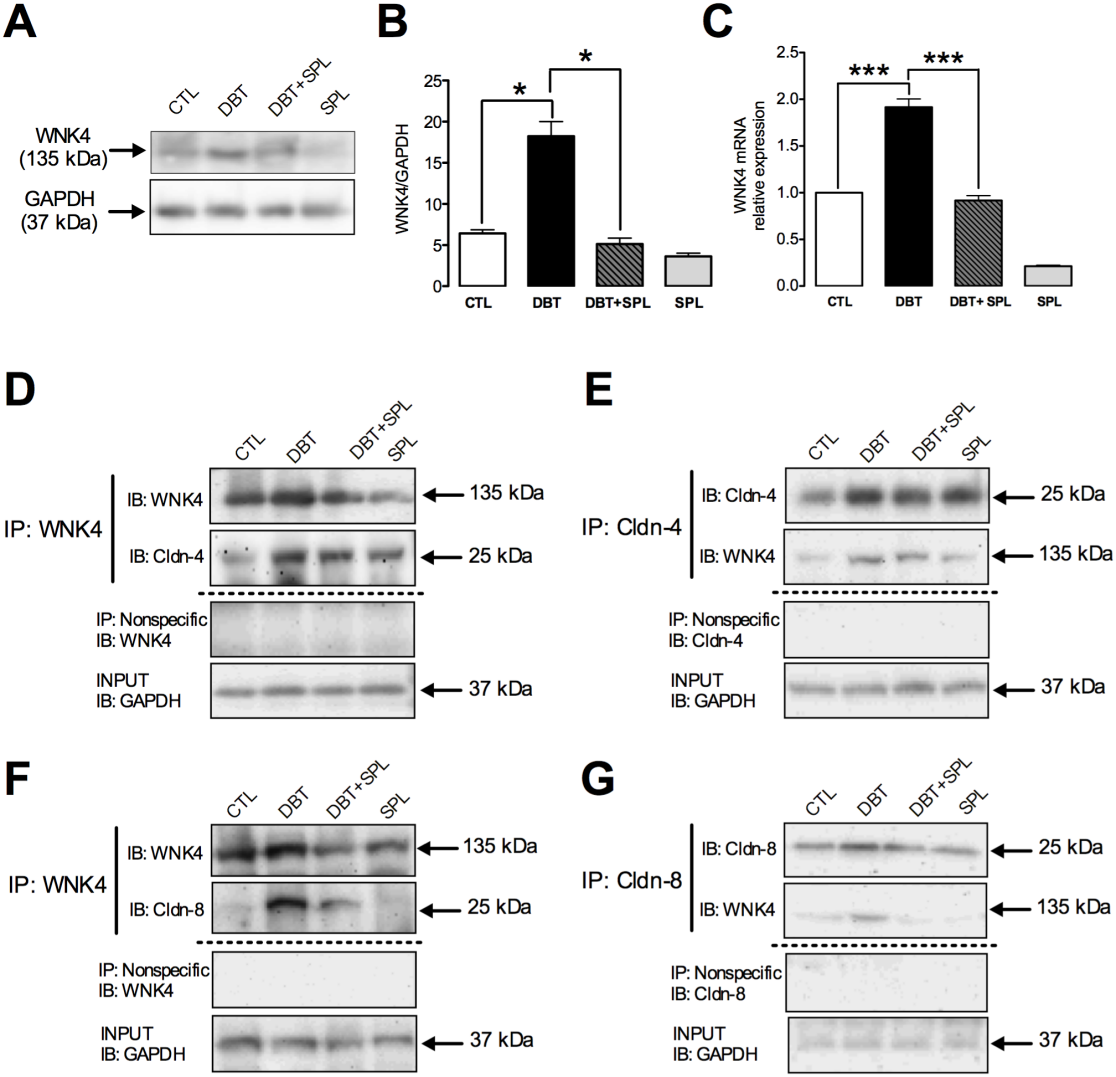
SPL treatment decreases diabetes-induced increased WNK4 expression and co-IP with cldn- 4 and -8. WNK4 expression was evaluated by IB and qRT-PCR in the four experimental groups (S6 Fig). As shown in panels A and C, diabetes induced WNK4 protein expression and mRNA levels, respectively. SPL treatment decreased these changes. Densitometric analysis of IB is shown in panel B. To evaluate direct interaction of WNK4 with cldn-4 and -8 at the TJ, co-IP analyzes were performed. As shown, diabetes induced co-IP of WNK4 with cldn-4 (D) and vice versa (E). Also, diabetic condition induced co-IP of WNK4 with cldn-8 (F) and vice versa (G). SPL treatment decreased these changes. As shown in panels D-G, no signal was found under nonspecific conditions of IP performed with an unrelated antibody. GAPDH was evaluated in isolated DT and input extract as loading control. Data are mean±SEM from 3 rats per group. *p<0.05 and ***p<0.001.

In the kidney, WNK4 is a serine/threonine kinase, localized at the TJs [39], which phosphorylates cldns, thus modulating their function [40–42]. Based on the evidence described above, we evaluated the co-localization of WNK4 with cldn-4 or -8, in the TJ by IF. It was found that diabetes increases the WNK4 label in both cytoplasm and intercellular junctions of DT, where a diffuse label is observed (Fig 6A and 6B, green color). This is consistent with previous findings were WNK4 label showed a similar pattern [39]. In the diabetic condition, the co-localization of WNK4 with cldn-4 or -8 was increased in TJ, respectively (Fig 6A and 6B, yellow color). However, this co-localization is not homogeneous in throughout TJ, raising the possibility that yellow coloration is the result of overlapping of labels between cldn-4 or -8 (red label) with WNK4 (green label). To confirm the direct interaction of these proteins, co-IP assays were performed. First, we evaluated the WNK4 expression by IB (Fig 7A), and qRT-PCR (Fig 7C) in isolated DT, respectively. It was found that diabetes induces mRNA and protein expression of WNK4, and SPL treatment significantly prevented these changes (Fig 7B and 7C). To evaluate whether WNK4 interacts with cldn-4 and -8, co-IP assays were performed. It was found that diabetes increased co-IP of WNK4 with cldn-4 (Fig 7D); these findings were confirmed by reverse IP (Fig 7E). Furthermore, co-IP of WNK4 with cldn-8 was also tested. In a similar way as happened with cldn-4, WNK4 co-immunoprecipitated with cldn-8 (Fig 7F), which was also confirmed by inverse co-IP (Fig 7G), and these changes were prevented by SPL treatment. These results demonstrated that diabetes induces the localization of WNK4 in the TJ, increasing its interaction with cldn-4 and -8.

### SPL decreases diabetes-induced threonine phosphorylation of cldn-4 and -8

**Fig 8:**
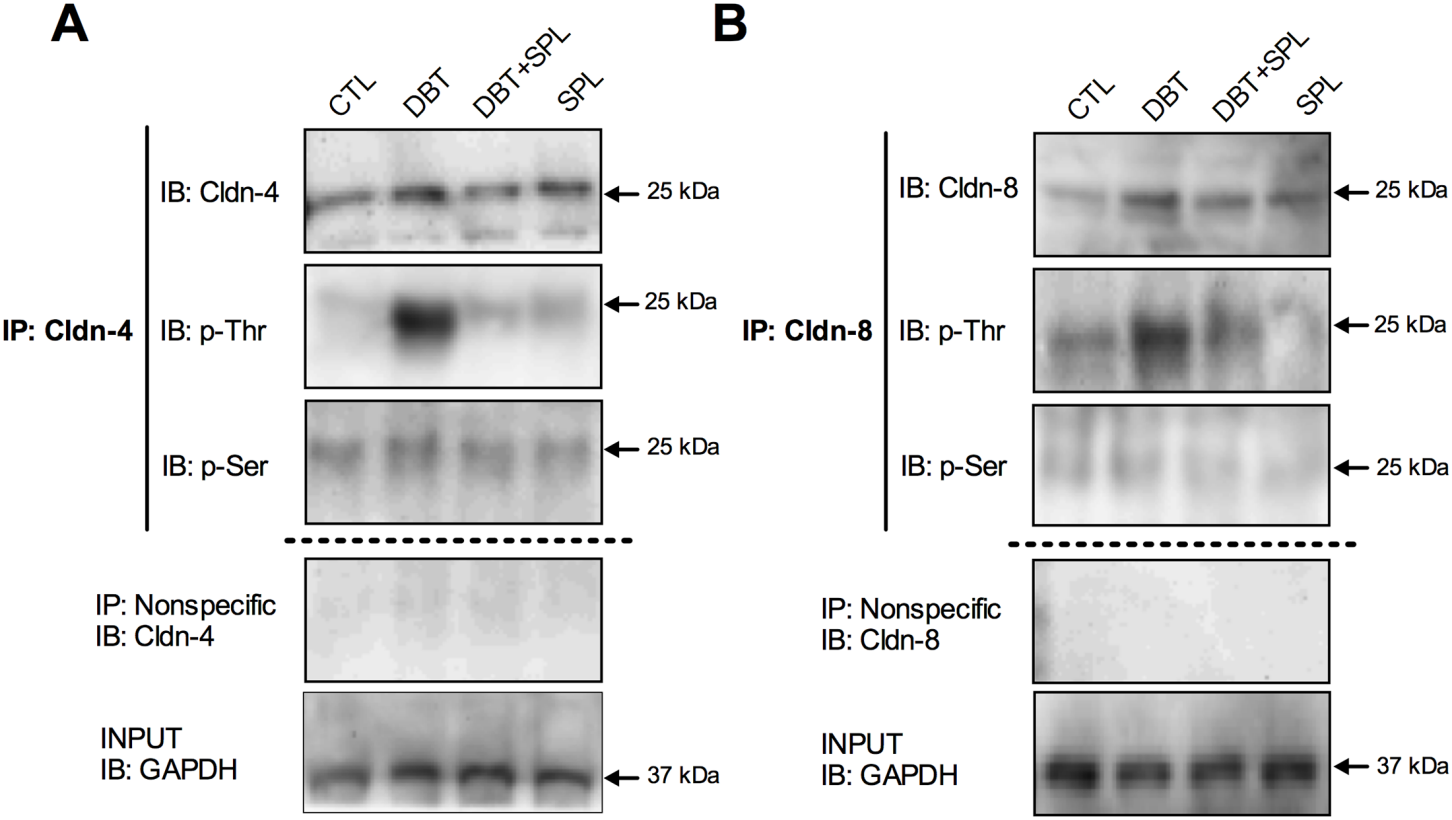
Diabetic condition induces phosphorylation of cldn-4 and -8 in threonine but not in serine residues. Phosphorylation of cldn-4 and -8 in threonine and serine residues was evaluated by IP assays in the four experimental groups. As shown, diabetes induced cldn-4 (A) and cldn-8 (B) phosphorylation in threonine, but not in serine residues. SPL treatment decreased these changes. As shown in panels A and B, no signal was found under nonspecific conditions of IP performed with an unrelated antibody. GAPDH was evaluated in input extract as loading control.

It has been described that ALD promotes rapid and transient phosphorylation of cldn-4 on threonine residues, in RCCD2 cells [43]. Moreover, it is known that cldns phosphorylation controls its localization and/or function [44]. Based on the findings above described, and that diabetes increase the interaction of WNK4 with cldn-4 and -8, we explored phosphorylation of cldn-4 (Fig 8A) and -8 (Fig 8B), in serine/threonine residues. Cldn-4 and -8 were immunoprecipitated from isolated DT, and phospho-serine and phospho-threonine antibodies were tested. It was found increased phosphorylation in threonine residues of cldn-4 (Fig 8A) and cldn-8 (Fig 8B), under diabetic condition, without changes in serine phosphorylation. SPL treatment decreased threonine phosphorylation of cldn-4 and -8. These findings showed that diabetes increased the threonine phosphorylation of cldn-4 and -8 mediated by WNK4 activity, which was decreased by SPL treatment.

### SPL decreases diabetes-induced SGK1 expression and its co-localization with WNK4

**Fig 9:**
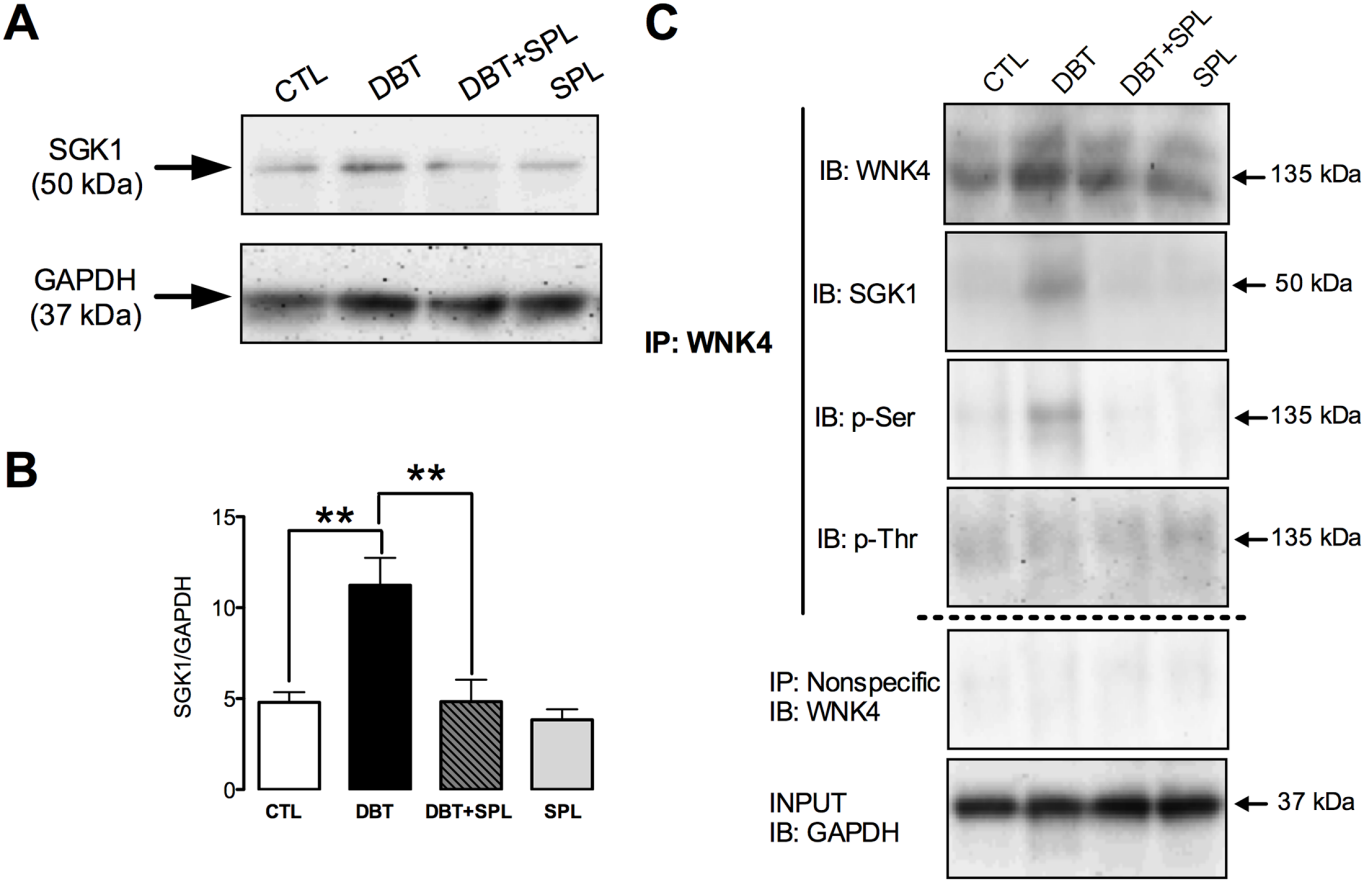
SPL treatment ameliorated diabetes-induced increased expression of SGK1, WNK4 and co-IP of WNK4 with SGK1. SGK1 expression was evaluated by IB analysis of isolated DT. As shown in panel A, DBT induced SGK1 expression. Densitometric analysis is shown in panel B (S7 Fig). Diabetic condition also induced co-IP of WNK4 with SGK1, and serine, but not threonine phosphorylation of WNK4 (C). SPL treatment prevented these changes. GAPDH was used as loading control in isolated DT and input extracts. As shown in panel C, no signal was found under nonspecific conditions of IP performed with an unrelated antibody. Data are mean±SEM from 3 rats per group. **p<0.01.

In the ASND, SGK1 is a major direct transcriptional target of ALD signaling, and of the activation of MR [45]. In mammalian kidney cells, SGK1 interacts with WNK4, forming a physical complex [46]. SGK1 phosphorylates WNK4 at serine 1169 promoting K^+^ secretion [46]. To further corroborate whether diabetes alters the phosphorylation of WNK4 by SGK1, we analyzed the SGK1 expression in the four experimental groups and found that diabetes induces SGK1 expression (Fig 9A) and SPL significantly prevented these changes (Fig 9B). Also, co-IP assays showed that diabetes induces the interaction of SGK1 with WNK4, which was prevented by SPL (Fig 9C). Furthermore, phosphorylation of WNK4 was analyzed and it was found that diabetes increases serine but not threonine phosphorylation of WNK4. These changes were also prevented by SPL treatment. Taken together, these findings demonstrate that diabetes increases WNK4 phosphorylation at serine residues mediated by SGK1 activity.

## Discussion

The ASDN is responsible of the reabsorption of approximately 10% of filtered NaCl by the GL, thus regulating blood pressure and extracellular fluid homeostasis. Also, ALD regulates extracellular fluid volume, and Na^+^ and K^+^ metabolism through activation of the MR. These effects are mediated by the activation of the transcellular transporters: renal outer medullary K^+^ channel (ROMK), the apical epithelial sodium channel (ENaC), the basolateral Na^+^/K^+^ ATPase pump and the sodium chloride cotransporter (NCC) [47–50]. Some *in vitr*o studies have suggested that ALD might modulate the paracellular pathway, mediated by cldns, by regulating phosphorylation of cldn-4 in cultured rat cortical collecting duct cells [43], and transcription of cldn-8 in distal colon [38]. In addition, it is widely known that ALD also promotes tissue injury by inducing inflammation, oxidative stress and fibrosis, and participates in the development and progression of renal complications in diabetes [18,19].

In our previous studies, we found that diabetes decreases the expression of cldn-5 in GL and cldn-2 and occldn in PT, and induces the expression of cldn-4 and -8 in DT [9]. In our model of early diabetic nephropathy, characterized by increased proteinuria and natriuresis without changes in serum creatinine and creatinine clearance, we found increased serum level of ALD in the STZ- and STZ+SPL groups. These data are in agreement with previous studies showing that diabetes increases renal cortical and total kidney CYP11B2, a key enzyme for ALD synthesis, mRNA and protein expressions, thus suggesting that also ALD might be produced locally in the kidney under diabetic conditions [51].

Based on the findings above described, we aimed to test whether ALD regulates *in vivo* cldns and occldn expressions by using SPL. As shown, SPL treatment exerted nephroprotection by decreasing diabetes-induced loss of body weight, proteinuria, proteinuria/creatininuria ratio, natriuresis and FENa^+^ without effect on blood glucose concentration. These dates were confirmed by analysis of WT1 and nephrin (glomerular markers of damage) and Kim-1 and Hsp72 (markers of proximal tubular damage). The results described herein showed new mechanisms through which blockade of ALD receptors depicted beneficial effects on glomerular lesions observed in models of type 1 diabetes and provide additional information to previous reports [23,24,36,52]. Also, SPL has been shown to prevent diabetic renal injury by decreasing oxidative stress, inflammation, apoptosis and fibrosis [23–28].

The natriuresis associated to diabetes may not be dependent on the actions of ALD in the ASDN, since under these circumstances decreased natriuresis would be expected. Nevertheless, we have previously reported that increased natriuresis is an early alteration observed in diabetic nephropathy and that this is due to loss of cldn-2 in proximal tubule [9]. Additionally, we have showed that all-trans retinoic acid preserves cldn-2 expression under diabetic condition, ameliorating natriuresis [34], thus confirming that cldn-2 loss leads to increased natriuresis. These findings are in agreement with the results described herein, because SPL treatment also preserved cldn-2 expression in PT at mRNA and protein levels. These data may be associated with the effect of SPL on decreased oxidative stress in PT. In this respect, SPL also decreased diabetes-induced oxidative stress in GL which may be associated to the preservation of cldn-5 and decreased proteinuria found in the DBT+SPL group. It is interesting that diabetes-induced loss of cldn-5 protein is not related to its mRNA levels, because they were not altered. Similar results were found in occldn in the PT, where SPL also prevented diabetes-induced loss of this protein which may be related to SPL-induced decrement of oxidative stress in PT. However, similarly to cldn-5, mRNA levels of occldn were not altered under diabetic condition, thus suggesting that loss of both cldn-5 and occldn may be due to posttranslational modifications related to oxidative damage, as suggested in previous studies on oxidative modifications of cldn-2 in diabetes [9].

In DT, SPL diminished the increment in TJ localization, mRNA and protein expression of cldn-4 and -8 induced by diabetes, thus suggesting that ALD mediates the transcription and expression of cldn-4 and -8 under diabetic condition. Increment in the co-immunoprecipitation of both cldns observed in diabetes might be related with its overexpression. This is consistent with the findings that cldn-4 and -8, co-immunoprecipitate, co-traffic, and co-localize in epithelial cells, where they form a paracellular Cl^―^ channel [16]. Furthermore, cldn-8 recruits cldn-4 to the tight junction and in the absence of cldn-8, cldn-4 is confined to the endoplasmic reticulum and to the Golgi complex [16]. Additionally, in the distal colon it has been proposed that Na^+^ absorption is paralleled by cldn-8-mediated sealing of the paracellular barrier to prevent immediate back-leakage of freshly absorbed Na^+^ by ENaC [38]. Having this in mind, it is tempting to speculate that ALD stimulates renal Na^+^ transport at the distal nephron not only by increased transcellular reabsorption but, in parallel, also by tightening the paracellular pathway, through increments in cldns -4 and -8. Thus, ALD may play a significant role in limiting paracellular back-leakage of Na^+^ along the ASDN and thereby may enhance net Na^+^ reabsorption.

Phosphorylation has been implicated in control of cldns localization and/or function [44]. In this study, we found that diabetes induced threonine phosphorylation of cldn-4 and -8. This is in agreement with previous *in vitro* studies where cldn-4 is phosphorylated by ALD through WNK4 and WNK1 [40,53]. WNK4 is a serine/threonine kinase expressed in the distal nephron that regulates ion transporters or channels such as thiazide-sensitive NaCl cotransporter (NCC) or ROMK channel. WNK4 might also regulate paracellular Cl^―^ permeability. When overexpressed in cell lines, WNK4 [particularly in the pseudohypoaldosteronism, type II (PHAII) mutant] stimulates paracellular Cl^―^ conductance [40–42]. WNK4 phosphorylates the carboxyl-terminus of cldn-4 and alters paracellular Cl^―^ permeability in cultured MDCK cells [40]. Also, WNK4 and cldn-7 co-localize in renal tubules of rat kidneys and co-immunoprecipitate in kidney epithelial cells. In this context, WNK4 phosphorylates ser206 in the carboxyl-terminus of cldn-7, which significantly increased paracellular permeability of Cl^―^ [42]. In the kidney, WNK4 localization was described at the TJ of the distal convoluted tubule and collecting duct [39]. Herein we found that diabetes increases the TJ localization, transcription and protein level of WNK4. Also, it was found that diabetes promotes WNK4 co-localization with cldn-4 and -8 in the TJ, thus suggesting that WNK4 interacts directly and phosphorylates cldn-4 and -8 on threonine residues. These effects were decreased by SPL, suggesting that MR activation induces the expression and TJ localization of WNK4 and in consequence promotes the phosphorylation of cldn-4 and—8 mediated by this kinase.

Moreover, SGK1 is a major direct transcriptional target of ALD signaling and of the activated MR [45]. Diabetes increased SGK1 expression and induced its co-immunoprecipitation with WNK4. This is in agreement with previous findings that describe that SGK1 can exist in a physical complex with WNK4 in mammalian kidney cells and that SGK1 phosphorylates WNK4 at serine 1169 [46]. In this state, ALD increases SGK1-induced WNK4 phosphorylation and promotes K^+^ secretion. Herein it was found that diabetes induced serine phosphorylation of WNK4. The findings above described suggest that SGK1 might be the kinase that phosphorylates WNK4, which is reinforced by the increased co-immunoprecipitation of both proteins under diabetic condition, and their decreased interaction in the SPL-treated rats.

In summary, although ALD increases Na^+^ reabsorption in the ASDN under diabetic condition (mediated by the sealing or the paracellular route by cldn-4 and -8), the general balance favours increased natriuresis. These findings tempt us to speculate that decreased reabsorption of Na^+^ in the PT due to loss of cldn-2 is not compensated in the ASDN, leading to Na^+^ loss in urine, observed in early stages of diabetic renal damage, before establishment of renal failure.

## Conclusion

**Fig 10:**
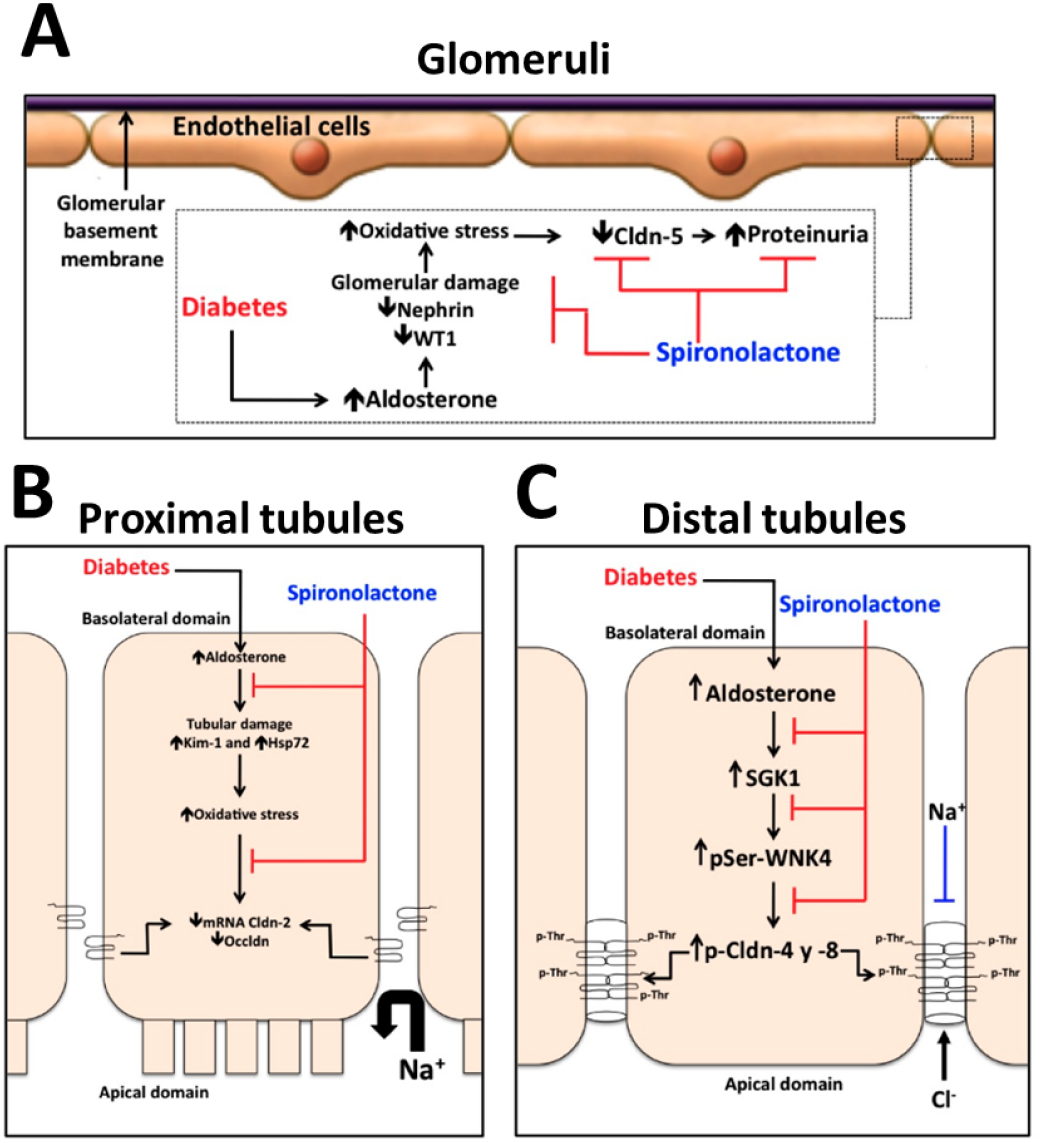
Schematic representation of the mechanisms involved in ALD-induced decrement of expression of cldn-5 in GL and, cldn-2 and occldn in PT, and increased expression of cldn-4 and -8 in DT. (A) In GL, diabetes-induced ALD promotes glomerular damage, evaluated by decreased expression of nephrin and WT1, and oxidative stress, which may be associated to decreased cldn-5 expression and proteinuria. (B) In PT, diabetes-induced ALD promotes tubular damage, evaluated by increased expression of Kim-1 and Hsp72, and oxidative stress, which may be associated to decrements of occldn and cldn-2 expressions, this latter may explain increased natriuresis. (C) In DT, diabetes-induced ALD induces the expression of SGK1, which in turn phosphorylates serine (pSer) residues of WNK4 promoting its localization in the TJ. This latter phosphorylates threonine residues (pThr) of cldn-4 and -8, where both proteins constitute a paracellular Cl^―^ channel and a Na^+^ barrier. In all nephron segments, blockade of ALD actions with SPL decreased oxidative stress in GL and PT and the expression, and phosphorylation of cldn-4 and -8 in DT.

In this study, we report for the first time that ALD mediates the decreased expression of cldn-5 in GL and cldn-2 and occldn in PT associated to oxidative stress, and the increased expression and phosphorylation of cldn-4 and -8 by a SGK1 and WNK4 signaling pathway in early stages of diabetic nephropathy, this was confirmed by blocking ALD actions with SPL. An integrative scheme of these mechanisms in the different nephron segments is shown in Fig 10.

## Supporting information

S1 Fig Data used for the plotting of histograms of the western blot in the Fig 1. Raw data used to densitometric analysis of nephrin/GAPDH (D), WT1/GAPDH (E), Kim-1/GAPDH (G) and Hsp72/GAPDH (H), respectively.
(XLSX)

S2 Fig Data used for the plotting of histograms of the western blot and mRNA PCR in the Fig 2. Raw data used to densitometric analysis of cldn-5/GAPDH (B), cldn-2/GAPDH (C) and occldn/GAPDH (D), and to mRNA relative expression of cldn-5 (E), cldn-2 (F) and occldn (G), respectively.
(XLSX)

S3 Fig Data used for the plotting of histograms of superoxide anion production, lipid peroxidation, protein carbonylation and reduced glutathione (GSH) content in the Fig 3. Raw data used to densitometric analysis of superoxide anion production, lipid peroxidation, protein carbonylation and reduced GSH content in GL (A, B, C and D, respectively), and PT (E, F, G and H, respectively).
(XLSX)

S4 Fig Data used for the plotting of histograms of the western blot and mRNA PCR in the Fig 4. Raw data used to densitometric analysis of cldn-4/GAPDH (C) and cldn-8/GAPDH (G), and to mRNA relative expression of cldn-4 (D) and cldn-8 (H), respectively.
(XLSX)

S5 Fig Data used for the plotting of histograms of the ratio cldn-4 and cldn-8 in the Fig 5. Raw data used to densitometric analysis of ratio between cldn-8/cldn-4 (B),and cldn-4/cldn-8 (D), respectively.
(XLSX)

S6 Fig Data used for the plotting of histograms of the western blot and mRNA PCR in the Fig 7. Raw data used to densitometric analysis of WNK4/GAPDH (B), and to mRNA relative expression of WNK4 (C), respectively.
(XLSX)

S7 Fig Data used for the plotting histograms of the western blot in the Fig 9. Raw data used to densitometric analysis of SGK1/GAPDH.
(XLSX)
